# Clinical, Immunological, and Genetic Findings in a Cohort of Patients with the DiGeorge Phenotype without 22q11.2 Deletion

**DOI:** 10.3390/jcm11072025

**Published:** 2022-04-05

**Authors:** Antonino Maria Quintilio Alberio, Annalisa Legitimo, Veronica Bertini, Giampiero I. Baroncelli, Giorgio Costagliola, Angelo Valetto, Rita Consolini

**Affiliations:** 1 Pediatrics Unit, Department of Clinical and Experimental Medicine, University of Pisa, 56126 Pisa, Italy; antonino.alberio@gmail.com (A.M.Q.A.); g.baroncelli@med.unipi.it (G.I.B.); giorgio.costagliola@hotmail.com (G.C.); 2 Section of Clinical and Laboratory Immunology, Pediatric Unit, Department of Clinical and Experimental Medicine, University of Pisa, 56126 Pisa, Italy; a.legitimo@ao-pisa.toscana.it; 3 Section of Cytogenetics, Department of Laboratory Medicine, Azienda Ospedaliero-Universitaria Pisana, 56126 Pisa, Italy; v.bertini@ao-pisa.toscana.it (V.B.); a.valetto@ao-pisa.toscana.it (A.V.)

**Keywords:** DiGeorge syndrome, 22q11.2 deletion, thymic output, dendritic cells, immunodeficiency, autoimmunity, copy number variations, array-CGH

## Abstract

Chromosome 22q11.2 deletion syndrome (22q11.2DS) is a primary immunodeficiency characterized by a broad and heterogeneous clinical presentation associated with various degrees of T-cell deficiency. We report the clinical, immunologic, and genetic findings of a cohort of eight patients presenting with a clinical phenotype that is highly suggestive of this syndrome but without the 22q11.2 deletion. The cardinal features of 22q11.2DS, such as congenital heart defects, hypoparathyroidism, and facial dysmorphisms, were observed in the majority of the patient cohort. The unusual features are described in detail. The immunologic assessment showed various degrees of immunodeficiency of the T-cell compartment, notably a reduction in the thymic output. Half of the patient cohort exhibited a reduction in total dendritic cells. Array comparative genomic hybridization (CGH) revealed six patients harboring copy number variations (CNVs) never reported in normal subjects. The gene content of these CNVs was carefully analyzed to understand the mechanisms leading to 22q11.2DS phenocopies. According to these results, we suggested that array-CGH should be used as a first-tier tool for patients resembling 22q11.2DS.

## 1. Introduction

The 22q11.2 genomic region is prone to meiotic errors due to the presence of several large blocks of low-copy repeats (LCRs) [1,2]. 22q11.2 deletion is causative of DiGeorge syndrome (DGS) and other clinical conditions, previously described separately, such as velocardiofacial syndrome (MIM #192430), conotruncal anomaly face syndrome (MIM #217095) (or Takao syndrome), (CTAFS), Opitz G/BBB syndrome (MIM #145410), and Cayler cardiofacial syndromes. All these conditions are now collected under the definition of “22q11.2 deletion syndrome” (22q11.2DS) according to the common genetic etiology [3]. 22q11.2DS has an estimated incidence of 1:4000 live births, with approximately 80–90% of cases presenting with de novo inheritance [2]. The 22q11.2 typical deleted region is approximately 3 Mb in size and harbors more than 40 protein-coding genes, seven microRNAs (miRNAs), and ten non-coding RNAs (according to build GRCh37). Different sets of genes are involved, such as *TBX1*, *HIRA*, and *COMT* [3], showing the great phenotypic variability that makes this pathology a classic example of a syndrome with variable expressivity and incomplete penetrance. The clinical phenotype is mainly characterized by congenital heart disease (CHD), palatal and craniofacial abnormalities, hypoparathyroidism, immune deficiencies or autoimmune diseases (related to thymic a/hypoplasia), and neurocognitive impairment [4,5]. The severity of symptoms is also variable, ranging from quite severe to near-normal life conditions [6]. After the introduction of array comparative genomic hybridization (CGH) technology, further copy number variations (CNVs) have been identified, which are associated with clinical pictures resembling 22q11.2DS [7,8,9,10]. However, in 6% to 17% of patients, the identification of a genetic cause remains unknown, with serious consequences for their therapeutic management.

In this paper, we describe the clinical picture, the immunological abnormalities, and the genomic alterations of a cohort of patients with highly evocative DGS phenotype without 22q11.2 deletion. This may contribute to the diagnosis of patients presenting with primary immunodeficiency and developmental defects of unknown etiology.

## 2. Materials and Methods

### 2.1. Study Design and Data Collection

We enrolled eight patients (four females and four males), who were followed at a single pediatric center for primary immunodeficiency (at the University of Pisa), presenting with a highly evocative clinical phenotype for 22q11.2DS. The Tobias criteria were used to consider these patients as susceptible to genetic analysis for 22q11.2DS [11]. The study was conducted according to the Declaration of Helsinki II. Informed consent was signed prior to performing the genetic analyses. Written and informed consent to report the clinical data and the publication of the genetic analysis was obtained from all patients’ parents or legal guardians. Patient data were retrospectively retrieved from the clinical records and anonymously entered into a database. The cohort of patients was composed of six children and two adults currently followed in our center. Physical phenotypes, including auxologic features, behavioral or psychiatric disorders, immunological profile, and genomic analysis were evaluated. Frequent morbidity was reported using recurrent respiratory infections (RRIs), according to the previously described RRI criteria [12]. The analysis of 25-hydroxyvitamin D (25OHD) levels was limited to patients who did not initially receive vitamin D supplementation; 25OHD levels were considered deficient for values < 20 ng/mL, according to the Institute of Medicine (IOM), the American Academy of Pediatrics (AAP), and the European Society for Paediatric Gastroenterology, Hepatology, and Nutrition (ESPGHAN) recommendations [13,14,15]. Auxological parameters of weight and height were expressed in standard deviation (SD) scores, using growth charts as previously described [16]. The measurements of height were performed at time points T0, T1, and T2; T1 and T2 were related to the measurement of the height after 2 and 4 years of follow-up, respectively.

### 2.2. Flow Cytometry and Immunological Assessment

None of the patients had acute infections at the time of sample collection for the immunological evaluation. Lymphocyte counts, serum immunoglobulin concentration, and serum immunoglobulin subclasses were evaluated through standard methods and compared with age-related normal values. An extended immunological phenotype was performed in all patients and the data were compared with age-matched normal values [17,18,19,20]. Eight-color flow cytometric analysis was performed on fresh peripheral whole blood anti-coagulated with ethylenediaminetetracetic acid (EDTA), according to standard protocols, to determine the following cell subpopulations: T lymphocytes (CD3^+^), helper T lymphocytes (CD3^+^CD4^+^), cytotoxic T lymphocytes (CD3^+^CD8^+^), B lymphocytes (CD19^+^), and natural killer (NK) cells (CD16^+^/56^+^). Helper and cytotoxic T lymphocytes were also analyzed for the expressions of CD45RA, CD62L, and CD31 to identify naïve (CD45RA^+^CD62L^+^), central memory (CM: CD45RA^−^CD62L^+^), effector memory (EM: CD45RA^−^CD62L^−^), terminal effector memory re-expressing CD45RA (TEMRA: CD45RA^+^CD62L^−^), and recent thymic emigrants (RTEs: CD45RA^+^CD62L^+^CD31^+^) [21]. Circulating Treg cells were identified as a CD4^+^CD25^+^CD127^−^ cell population, as previously described [22]. The expression of CD45RA was evaluated to estimate the amount of naïve Treg cells. The expression of CD185 (CXCR5) was analyzed on memory T helper cells (CD3^+^CD4^+^CD45RO^+^) to identify follicular T cells. We defined naïve B cells as CD19^+^CD27^−^IgD^+^ and switched memory B cells as CD19^+^CD27^+^IgD^−^IgM^−^. Circulating dendritic cells (DCs) were enumerated and phenotypically characterized directly into the two major subsets, namely, myeloid (mDCs) and plasmacytoid (pDCs), as previously described [22]. Due to the lack of a specific marker to detect DCs, we used a mixture of monoclonal antibodies specifically established to identify DCs, purchased from Immunotech (Beckman Coulter Inc., Brea, CA, USA). Cells were stained with the following antibodies: CD14, CD16, CD85k, CD33, or CD123 for the mDC and pDC subsets, respectively. Dendritic cells were identified as CD14^low/−^CD16^low/−^CD85k^+^ and CD33^+^ or CD123^+^. The absolute numbers of DCs were estimated by multiplying the percentage of DCs in the mononuclear cell (MNC) gate by the absolute peripheral blood MNC count determined using a standard hemocytometer (Abbott Laboratories, Abbott Park, IL, USA). DC data were compared with our laboratory age-related normal values [22]. Data acquisition and analysis were performed on a dual laser BD FACSCanto (Becton Dickinson Immunocytometry Systems, San Jose, CA, USA) using the FACSDiva software (San Jose, CA, USA).

### 2.3. Genomic Analysis

Karyotyping was performed according to standard methods. The commercially available D22S75/N25 probe (Cytocell, Cambridge, UK) was used, according to the manufacturer’s instructions, to perform fluorescent in situ hybridization (FISH) analysis.

Genomic DNA of the patients (tests) was isolated from peripheral blood using standard methods; DNA from healthy subjects (reference) was used as controls (Agilent Technologies, Santa Clara, CA, USA); tests and reference DNA were differentially labeled with Cy5-dCTP or with Cy3-dCTP using random primer labeling and applied to 60K arrays, according to the manufacturer’s protocol (Agilent, Santa Clara, CA, USA). Quality slide evaluation was performed using the Agilent dedicated software (Feature Extraction, Agilent). We elaborated only on those experiments that met the “excellent” criteria, as determined by the QC report (Cytogenomic software, Agilent). In particular, the derivative log ratio spread (DLRS) was the main value considered for further analysis of the data: when >0.16, the experiment was discarded and repeated. CNVs were identified with Cytogenomics 4.0.3.12 (Agilent) using the ADM-2 (aberration detection method 2) algorithm. The threshold was set to a minimum of 6 with the minimum number of 3 probes required in a region and a minimum absolute log ratio of 0.25. We analyzed all the CNVs with 3 or more contiguous probes for deletions and duplications. The CNVs reported in the Database of Genomic Variants (http://projects.tcag.ca/variation/ (accessed on 25 February 2022)) were classified as benign and not further analyzed. All the other genomic imbalances were compared with those collected in DECIPHER (https://decipher.sanger.ac.uk/ (accessed on 25 February 2022)) and ClinVar (https://www.ncbi.nlm.nih.gov/clinvar/ (accessed on 25 February 2022)). Data about the biological function of genes and their interactions were retrieved from UCSC Genome Browser (http://genome.ucsc.edu/ (accessed on 25 February 2022)), PubMed (https://www.ncbi.nlm.nih.gov/pubmed/ (accessed on 25 February 2022)), and OMIM (https://www.omim.org/ (accessed on 25 February 2022)).

## 3. Results

### 3.1. Patients Characteristics

Two patients (25%) met one of the A Tobias criteria, while six patients (75%) met at least two of the B Tobias criteria, along with a combination of C criteria. The mean age of patients was 6.7 years (8 months–15.7 years) at diagnosis and 13.7 years (2.2–23.7 years) at the time of the study. The mean follow-up time of the cohort was 55.2 months (SD ± 31.6). Demographic and clinical features of the cohort are described in Table 1. No exposure to tobacco, alcohol, or teratogenic drugs during pregnancy was reported; no perinatal information was available for the two sisters P4 and P5. Patient P2 was born from an emergency cesarean section at 32 weeks of gestational age for maternal HELLP (hemolysis, elevated liver enzymes, low platelets) syndrome. Subject P6 was born to a human immunodeficiency virus (HIV)-positive mother, small for gestational age, and successfully received the prevention protocol of vertical transmission. The overall cases were sporadic, without familial history of severe chronic diseases, immunodeficiencies, or inherited pathologic conditions. Subject P2 presented with syndactyly like her maternal grandmother. The neonatal period of subject P1 was complicated by seizures, hypotonia, and sucking difficulty. Congenital heart defects were detected in 87.5% of the cohort (*n* = 7); two conotruncal anomalies and four non-conotruncal defects (patent oval foramen, patent ductus arteriosus, atrial and/or ventricular septum) were observed. A total of 71% of them underwent corrective or palliative cardiac surgery in the first year of life, with excellent outcomes. Subject P3 presented with persistent left superior vena cava and percutaneous cardiac catheterization was performed at 5 months for aortic re-coarctation, with an absence of residual obstruction. Otolaryngologic malformations were detected and successfully corrected in two patients (25%). The overall cohort exhibited mostly mild facial dysmorphisms. Only toddler P8 presented with a gastrointestinal malformation (esophageal atresia). Concerning congenital renal disorders, medullary sponge kidney was found in subject P4 and hypospadias in P8. Noteworthy, language disorder with speech delay was observed in six subjects (75%) and psychomotor delay in 25%. One patient (P7) suffered from attention-deficit hyperactivity disorder, mixed anxiety disorder with an obsessive-compulsive component, and sleep disturbance; her nuclear magnetic resonance (NMR) showed widening of the fourth ventricle associated with hypoplasia of the lower cerebellar vermis. Interestingly, psychiatric involvement in patient P3 developed after many years from neurological manifestations. A total of three patients (37.5%) had hypoacusia that was conductive in nature, with a hearing aid being necessary only in one patient (P7). The two sisters P4 and P5 suffered from monthly headache episodes. Other anomalies, such as myopia (P4 and P6) and hypermetropia (P5, P6, and P7), were found in 25% and 37.5% of patients, respectively. Moreover, subject P6 showed left cryptorchidism and developed a limitation in pronation movement of the right upper limb in the last year of follow-up, which is still under diagnostic investigation.

### 3.2. Infections and Autoimmunity

Recurrent upper respiratory infections were detected in the majority of the cohort, including otitis and sinusitis (75%); lower respiratory tract infections occurred in two patients (25%), requiring hospitalization. No patient developed bronchiectasis. A total of 37.5% (three out of eight subjects) were affected by recurrent periodic fevers, without genetic features of monogenic autoinflammatory syndromes, and subject P2 was affected by recurrent urinary infections. Subjects P2 and P3 presented with recurrent herpes simplex infections and aphthous stomatitis. No fungal or opportunistic infections were identified during the follow-up, except for one episode of oral candidiasis in patient P2, requiring fluconazole prophylaxis. Regarding severe infections, an episode of mediastinitis in the context of the sternal surgical wound was described in child P8, with a good outcome.

Concerning autoimmune manifestations, juvenile idiopathic arthritis with oligoarticular phenotype was diagnosed in patient P3. Joint involvement (the right knee, right temporomandibular, and proximal interphalangeal of the fifth finger of the right hand) required a step-up therapy with frequent evacuative and infiltrative (triamcinolone acetonamide) arthrocentesis, use of modifiers of the biologic response (methotrexate), and biologic drugs (anti-tumor necrosis factors). Moreover, the child was unresponsive and developed a chronic inflammatory process that resulted in deformity of the hand joint and residual synovial inflammation of the right temporomandibular joint, as detected using nuclear magnetic resonance, with functional limitation.

### 3.3. Auxological and Endocrine Features

Weight and head circumference were within normal percentiles for the overall cohort, while a delay in height growth was remarked. No measurements were reported for subject P8 and, consequently, he was excluded. A reduction in the height (between −1 and −2 SD) was reported in almost all of the cohort, prevalently in childhood. No case of short stature (height < −2 SD) was registered. Of note, only the two sisters P4 and P5 presented within a height normal distribution (Supplementary Figure S1).

We focused on the endocrine features of the cohort by investigating the 25OHD level and its association with immunological parameters, as we previously published for a cohort of DGS patients [23]. Therefore, we identified three subgroups according to 25OHD levels: group A (normal values), group B (25OHD deficiency), and group C (patients with hypoparathyroidism). Toddler P8 was excluded due to the absence of 25OHD determination. Group B was composed of patients P2, P6, and P7. The vitamin D status was performed at diagnosis and showed the following 25OHD mean values: 30.23 ng/mL, 17.43 ng/mL, and 14.0 ng/mL in the A, B, and C groups, respectively (normal range < 20 ng/mL). Both the A and B groups presented with parameters of phospho-calcium metabolism (parathyroid hormone, PTH, calcium, phosphate, alkaline phosphatase) in the normal range for the patients’ ages. Patients belonging to group B received oral supplementation with cholecalciferol at the dosage of 25,000 IU/month. An increase in 25OHD values was observed in P2 (46.9 ng/mL vs. basal value 16.8 ng/mL) and P6 (35.5 ng/mL vs. basal value 17.63 ng/mL).

Patients P4 and P5 (25%) received a diagnosis of congenital hypoparathyroidism. Both presented with neonatal hypocalcemia, elevated serum phosphorus concentration, PTH values constantly suppressed or undetectable (<3 ng/mL), and low vitamin D values (13 ng/mL and 15 ng/mL in P4 and P5 patients, respectively). They received treatment with calcium carbonate (50–100 mg/kg/day) and calcitriol (20–40 ng/kg/day) for at least 6 years, with good outcomes. Moreover, the vitamin D values returned to within the normal range only for a short time and subject P4 suffered from two hypercalcemia episodes during the follow-up.

However, we did not observe any difference between vitamin D levels and the immunological parameters examined in the various groups.

### 3.4. Immunological Features

The lymphocyte and lymphocyte subpopulation values of patients, compared with age-matched normal controls, are shown in Table 2 and Table 3. Lymphocytopenia was reported in 62.5% of the cohort (five out of eight patients). A low absolute count of CD3^+^ lymphocytes and CD4^+^ and CD8^+^ cells were observed in 75% (*n* = 6), 87.5% (*n* = 7), and 75% (*n* = 6) of subjects, respectively. The B cell absolute number was low in two lymphopenic patients, with them being normal if expressed as a percentage. Similarly, the NK cell count exhibited the same behavior in patient P2; conversely, high relative numbers of NK cells were observed in P3, P6, P7, and P8. Furthermore, extensive phenotyping was performed due to the suspicion of a putative thymic a/hypoplasia. RTEs were lower than normal age-matched values in six out of eight patients, in particular, P1, P2, and P3 < 15%. Naïve helper T cells were reduced in P2 and severely low in both patients P1 and P3 (8.8% and 12.7%, respectively). Naïve cytotoxic T cells were reduced in two subjects (P3, P6). Central memory cytotoxic T cells were low only in P8, whereas they were higher than the normal range in P1, P6, and P7. Both effector memory CD8^+^ T cells (EM and TEMRA) were reduced in patients P5 and P7, while EM was only low in P4 and TEMRA only in P1. The overall cohort showed normal values of follicular T cells. Regulatory T cells were increased in patient P2 and reduced in patient P6. The analysis of the B cell compartment (naïve and switched memory cells) revealed mostly normal values, with the exception of two patients who showed a reduction of naïve B cells (P6 and P7) and an increase of switched memory cells (P6).

To explore the interface between innate and adaptative immunity, we investigated the dendritic cells, as shown in Table 4. A total of 50% of patients (P2, P3, P5, P7) exhibited a reduction in total DCs in comparison to healthy controls, whereas an increase was observed in patient P8. The observed reduction affected mainly the myeloid subset (37.5%), except for patient P2, who showed a reduction in both subsets.

Serum immunoglobulin levels are shown in Table 5. Two children (P2 and P6) had partial selective IgM deficiency (between −1 and −2 SD). Patients P3 and P5 (25% of the cohort) had a reduction in both IgG and IgA, with the IgA values being severely low (>−2 SD) in P5, whereas in P8, a reduction in both IgG and IgM (between −1 and −2 SD) was observed. P4 showed hypogammaglobulinemia (both IgG and IgA, between −2 and −3 SD), along with a compensatory increase (>+3 SD) in IgM levels, requiring immunoglobulin replacement therapy. Conversely, P1 had an increase in IgM (>+2 SD). IgE levels were normal in the overall cohort. The immunoglobulin subclass levels were in the normal range in the overall population, except for the IgG3 subtype, which was reduced in patient P8.

None of the patients had neutropenia. Mild eosinophilia (760/µL) was observed in subject P3.

### 3.5. Genomic Features

The genetic assessment of all patients did not show any deletion in the 22q11.2 and 10p13-14 regions. Among the eight patients tested using array-CGH, two subjects did not show any pathological CNVs, according to the DGV database (http://dgv.tcag.ca/variation (accessed on 25 February 2022)), which collects variations reported in normal subjects. Table 6 shows the CNVs detected in the remaining six patients, along with the positions of the first and last abnormal probes and the extent and gene content of each CNV. The pattern of inheritance was assessed in four patients in which CNVs were inherited from the mother.

Patient P1 showed a duplication of 314 kb in 2q24.1, inherited from her mother, that harbored the long intergenic non-protein coding RNA 1876. No overlapping duplications were reported in the Decipher database (https://decipher.sanger.ac.uk/ (accessed on 25 February 2022)), with this CNV classified as a variant of unknown significance (VOUS).

P4 and P5 were two sisters who showed a duplication in 20p11.22 inherited from their mother. This CNV has never been reported in individuals with a pathological phenotype (Decipher). This region harbors two non-coding protein LINC01727, LINC01726 and two coding protein PAX1 and NKX2 genes. PAX1 (*167411) encodes a transcription factor implicated in embryogenesis in vertebrates and plays an important role in segmental spine formation and thymus organogenesis. In humans, the phenotypic effects of the PAX1 duplication have not been yet described, whereas homozygous mutations of this gene have been associated with otofaciocervical syndrome 2 with T-cell deficiency (OTFCS2) (#615560) [24,25,26]. NKX2 contains a homeobox domain. It is highly expressed in the central nervous system and encodes for a protein that is likely a nuclear transcription factor involved in the morphogenesis of this system. According to these data, this CNV is likely pathogenetic.

In patient P6, two duplicated regions were detected in 11p15.5 and the pseudoautosomal region Xp22.33/Yp11.32. The duplication in 11p15.5 encompassed a total of 15 genes (Supplementary Table S1), among which, EPS8L2, TALD01, SLC25A22, PNPLA2, and CD151 are reported in the OMIM database (https://www.omim.org (accessed on 25 February 2022)). Apparently, none of these five genes appeared causative of the clinical features of this patient (Supplementary Table S1). Moreover, we highlight that the pathological phenotype described in OMIM is related to homozygous loss-of-function mutations. No overlapping duplications are reported neither in the literature nor in Decipher; thus, according to the few data available, this variant could be classified as VOUS. The duplication in Xp22.33/Yp11.32 harbors one non-coding gene (LINC00685) and four coding genes, including SHOX (*312865/*400020) (Supplementary Table S1). SHOX/SHOX enhancer deletions cause short stature and skeletal abnormalities (#249700, #127300, #300582); microduplications in the pseudoautosomal region including SHOX appear to be rare and have been related to autism spectrum disorders and neurodevelopmental pathologic conditions [27].

Patient P7 showed a deletion in 17q21.31 of about 493 kb that was causative of Koolen–De Vries syndrome (KDVS) (#610443). Moderate-to-severe intellectual disability, hypotonia, and a characteristic face represent the core phenotype of this syndrome. More variable features include cardiac, genitourinary anomalies, seizures, nasal speech, and a friendly demeanor [28]. Haploinsufficiency of KANSL1 (*612452) appears causative of the syndrome, as the clinical phenotype does not substantially differ between patients with 17q21.21 microdeletion encompassing KANSL1 and patients with a de novo heterozygous mutation in this gene. KANSL1 encodes a nuclear protein that plays a role in chromatin modification. It is a member of a histone acetyltransferase (HAT) complex [29].

Patient P8 showed two deleted regions in 17q13.2 and Xq24, with sizes of 258 kb and 68 kb, respectively, both inherited from his mother. The deletion in 17p13.2 harbors WSCD1, which encodes for a membrane protein with a sulfotransferase activity, whose role is not yet known. This CNV can be classified as a VOUS. The deletion in Xq24 encompasses two genes, namely, CXorf56 and UBE2A, and the phenotypic effects of their loss-of-function in males are reported in OMIM. CXorf56 is related to “Intellectual development disorder, X-linked 107” (#301013). UBE2A encodes a member of the E2 ubiquitin-conjugating enzyme family that is required for post-replicative DNA damage repair and may play a role in transcriptional regulation; its loss-of-function is causative of “UBE2A deficiency syndrome” or “X-linked Nascimento-type intellectual disability syndrome” (#300860). According to these data, this CNV is pathogenetic.

## 4. Discussion

We described the clinical phenotype of a cohort of eight patients that were highly suggestive of 22q11.2DS without harboring genomic aberrations of chromosome 22. The cardinal features of 22q11.2DS, such as congenital heart defects, hypoparathyroidism, facial dysmorphisms, and immunological abnormalities, were observed in this cohort of patients.

The typical cardiac defects, such as tetralogy of Fallot, right-sided aortic arch, and truncus arteriosus, were identified in a few patients of our cohort, whereas minor cardiac anomalies were observed with higher frequency and uncommon defects, such as aortic coarctation and hypoplastic left heart syndrome, were also described. Peculiar facial dysmorphic features, such as hypertelorism, narrow palpebral fissures, epicanthal folds, and micrognathia, were observed in all the patients of the study; furthermore, some uncommon traits, such as wide ear pad, low set ears, flat nasal bridge, and enlarged nasal, were also found. Hypoparathyroidism was not associated with cardiac defects, contrary to what is reported in 22q11.2DS. Concerning the neurodevelopmental disorders, they did not differ from 22q11.2DS, with speech and psychomotor delays being the most frequent manifestations identified during the follow-up. The frequency of the other phenotypic features was similar to that of typical 22q11.2DS [2,4,10,30].

The immunological profile did not substantially differ from typical 22q11.2DS, showing various degrees of mild or moderate immunodeficiency, mainly related to the cell-mediated compartment. We reported a reduced thymic output entailing low RTEs and reduced numbers of both CD4^+^ and CD8^+^ naïve T lymphocytes. The decline in naïve and the increase in memory T-cell populations observed in our cohort could have been due not only to the impaired thymic output but also to the accelerated conversion of naïve to memory phenotype, secondary to multiple mechanisms, such as infectious exposures or homeostatic expansion, as we previously reported [31]. No severe phenotype resembling a leaky SCID was observed in our cohort. Thymic hypoplasia and, more recently, the immature status of the thymus (mainly referred to as an impairment of the epithelium function) described in the majority of cases of 22q11.2DS [5,32] may have underlain the immunological abnormalities observed in our population. We previously described a defective Tregs number in 22q11.2DS [23] that, when associated with an impaired expression of AIRE-dependent tissue-restricted antigens, leads to an impaired peripheral tolerance and consequent escape of autoreactive T cells [32]. Conversely, the Tregs number was normal in our patients, as well as the number of switched memory B cells, which were reported (together with a low level of naïve T helper cells) as strong predictors for the development of autoimmune disorders in DGs patients, particularly in their adult life [33]. Interestingly, a low level of DCs was observed in half of our patients, as we recently reported in 22q11.2DS [23], most prevalently in the mDC compartment. We argue that this finding may contribute to the observed high susceptibility toward developing infectious diseases and autoimmune manifestations of these patients. Concerning vitamin D status, the limited population analyzed did not allow for investigating its relationship with the immunological parameters, as we previously reported in 22q11.2DS. Furthermore, due to the recognized immunomodulatory role of vitamin D, it appears reasonable to suggest its supplementation also in these patients, as infections and susceptibility to autoimmune diseases represent their major concern, similarly to 22q11.2DS.

Genetic assessment in our cohort revealed six patients harboring CNVs that are never reported in normal subjects (Table 6). The genes content of these CNVs was carefully analyzed for their correlation with the phenotypic features reminiscent of 22q11.2DS.

In subject P1, a 314 kb duplication in the 2p24.1 region, where the long intergenic non-protein coding RNA 1876 (LINC1876) is harbored, was identified. According to GeneHancer (https://genome.ucsc.edu (accessed on 25 February 2022)), LINC1876 regulates the expression of NR4A2, a gene located distally to the duplicated region. NR4A2 encodes a steroid–thyroid hormone-retinoid receptor, acting as a nuclear receptor (NR) transcription factor, and is mainly expressed in neurons of several areas of the CNS where it is specifically required for the development and function of the neurons. The dysregulation of this gene has been associated with neurodevelopmental delay and intellectual disability with or without epilepsy [34]. Altered NR4A2 expression is thought to have caused the neurological phenotype of P1, characterized by epilepsy with psychomotor delay and cognitive impairment. It was demonstrated that the NR4A family has a role in T cell development from thymic differentiation to peripheral response against infections and cancer; the overexpression of NR4A1 and NR4A3, but not NR4A2, induces thymocyte apoptosis in vivo [35]. Although NR4A2 appears not to be involved in this mechanism, its altered expression in our patient might contribute to determining his severe deficiency of thymic output.

P4 and P5 patients were two sisters who exhibited a 365 kb duplication in the 20p11.22 region, including the PAX1 gene. PAX1 is a member of the paired box (PAX) family of transcription factors that plays a critical role in human embryogenesis at the level of pharyngeal pouches, involving the development of the thymus, tonsils, parathyroid glands, thyroid, and middle ear [26,36,37]. PAX1 homozygous loss-of-function variants are causative of otofaciocervical syndrome 2 with T-cell deficiency (OTFCS2) (#615560 OMIM), which may include a severe combined immunodeficiency (SCID) due to abnormal thymic epithelium development [26]. The main aspects of this syndrome are facial anomalies, cup-shaped low-set ears, preauricular fistulas, hearing loss, branchial defects, skeletal anomalies, and mild intellectual disability [24,25]. Although the effects of PAX1 duplication are still unknown, it might alter the embryonic development of the pharyngeal region. It is possible to hypothesize that PAX1 dosage alteration can contribute specifically to the otolaryngological manifestations, such as the hearing loss observed in patient P5 and to the dysmorphic auricular appendix in patient P4, as well to the hypoparathyroidism and immunological alterations found in both sisters, which is usually observed in 22.11.2DS.

In patient P6, the coexistence of two CNVs, located on 11p15.5 and the pseudoautosomal regions Xp22.33/Yp11.32 was detected. The 11p15.5 duplication was rich in genes, five of which are related to known syndromes with an autosomal recessive inheritance (#617637, #606003, #609304, #610717, #609057 OMIM). The effects of their duplication are unknown, but, according to their expression and function, none of them seem to influence the patient’s phenotype.

The duplication in the pseudoautosomal region Xp22.33/Yp11.32 included SHOX, encoding a homeodomain transcription factor involved in cell cycle and growth regulation. SHOX deletions cause well-defined pathologic phenotypes, mainly including short stature and skeletal abnormalities (#249700, #127300, #300582 OMIM). Recently, it was highlighted that microdeletions encompassing this gene are a risk factor for autism spectrum disorders and neurodevelopmental defects [27]. We outline the possible role of this variant in determining the speech delay observed in the patient. In the human embryo, SHOX is expressed both in the limbs and in the first and second pharyngeal arches, from which originate the maxilla, mandible, and several bony elements of the external and middle ear [38]. It could be speculated that conductive hearing loss may be attributed to the dysregulation of this gene.

In P7, a 17q21.13 deletion was identified, which is causative of the Koolen–De Vries syndrome (KDVS #610443). KDVS has variable expressivity and a wide clinical spectrum that can overlap with DGS. The pathologic features of the patient include neurodevelopmental delay, anxiety disorder with psychotic signs, as well as facial dysmorphisms (long face, malar flatness, hooded eyelids resulting in the appearance of narrow palpebral fissures, and bulbous nasal tip), which overlap between both syndromes [39]. The other clinical features, such as skeletal anomalies and otolaryngological manifestations, are less common in KDVS. Interestingly, a patient with KDVS was also identified in a previously reported cohort of DGS without a 22q11.2 deletion [8]. The overlapping phenotypes of these two syndromes could be attributed to an underlying common genetic pathway. The master gene of KDVS is KANSL1, a protein-coding gene that belongs to a histone acetyltransferase (HAT) complex. Even if direct interactions between KANSL1 and the protein-coding genes located in the 22q11.2-deleted region have not been demonstrated, a common miRNA regulatory network has been identified. miRNAs play a role in 22q11.2DS [40]; interestingly, it was highlighted that specific miRNAs (such as miR-106b-5p, miR-148a-3p, miR-23b-3p, miR-17-5p, miR-149-5p, and miR-130b-3p) involved in the KANSL1 regulation also regulate DGCR14, DGCR2, TXNRD2, MRPL40, and CRKL genes, which are included in the 22q11.2-deleted region [41].

Patient P8 showed two deletions: the first in 17p13.2 harboring the WSCD1 gene and the second one in Xq24 encompassing the CXorf56 and UBE2A genes. The clinical effects of WSCD1 haploinsufficiency are unknown, whereas the deletion of CXorf56 and UBE2A was related to “Intellectual developmental disorder, X-linked 107” (#301013) and to “UBE2A deficiency syndrome” or “X-linked Nascimento-type intellectual disability syndrome” (#300860), respectively. Developmental delay, motor delay, impaired speech, and mild axial hypotonia, all present in P8, could be related to this Xq24 deletion. The phenotypic spectrum of UBE2A deficiency syndrome was recently expanded, and cardiac defects, craniofacial dysmorphisms, urogenital malformations, and hypogammaglobulinemia emerged as frequent features [42,43]. Therefore, the clinical spectrum of our patient, including cardiologic and urogenital defects and facial dysmorphic features, together with hypogammaglobulinemia, could be attributed to this deletion.

## 5. Conclusions

We outline that array-CGH should be used as a first-tier tool in the diagnostic work-up of patients presenting with a phenotype resembling the 22q11.2DS. This technique allows for identifying CNVs, whose altered gene content should be carefully examined to understand the mechanisms leading to 22q11.2DS phenocopies. Further analysis, such as whole exome sequencing and methylome analyses, could be considered in DGS patients in the case of normal array-CGH.

## Figures and Tables

**Table 1:** Clinical features of the patients.

P	Sex	Age at Diagnosis (Years)	Frequent Morbidity	Autoimmune Disorders	Cardiac Malformations	Otolaryngologic Involvement	Neuro-Behavioural and Psychiatric Involvement	Endocrine Involvement	Dysmorphic Features and Dental Issues	Skeletal Abnormalities
1	F	15.5	Ear infections and sinusitis	nr	VSD, ASD		Psychomotor and language delay, epilepsy, moderate cognitive impairment		Right eye exophoria	Left flat foot, cleft posterior arch in cervical vertebrae C1
2	M	1.7	Upper and lower respiratory tract infections, urinary infections	nr	VSD, ASD, CoA	Adenoid hypertrophy	Language delay, moderate cognitive impairment	25OHD deficiency	Long face, hypertelorism, low and flat nasal bridge, low and retracted ears	Syndactyly IV and V finger hands
3	M	7.7	nr	JIA	HLHS, CoA, BAV	Short lingual frenulum	Motor and language delay, anxiety disorder with an obsessive-compulsive component, vocal tics		Wide ear pad, supernumerary and ectopic teeth in the hard palate	Bilateral clinodactyly V finger, lumbar scoliosis with right dorsal hump, mild leg dysmetria, right leg hypotrophy, valgus right foot, mild retro-tibial torsion
4	F	10.5	Ear infections and sinusitis	Chronic autoimmune thyroiditis				Hypo-parathyroidism	Low set ears, preauricular appendix	
5	F	2.5	Ear infections and sinusitis	nr	PFO	Conductive hearing loss		Hypo-parathyroidism	Low set ears	
6	M	0.8	Upper respiratory tract infections	nr	TOF	Conductive hearing loss, adenoid hypertrophy	Language delay	25OHD deficiency	Anteroverse ears, bilateral epicanthal folds	
7	F	13.4	Upper and lower respiratory tract infections, urinary infections	nr	PDA	Labiopalatoschisis, mild and predominantly conductive mixed hearing loss	Psychomotor, cognitive and language delay, attention-deficit hyperactivity disorder, mixed anxiety disorder with an obsessive-compulsive component, sleep disturbance	25OHD deficiency	Eyes with elongated and upward rhymes, pyriform aspect of the nose with prominent tip and widened nostrils, lower lip with a thickened edge, wide ear pad with an antiverse and low implantation	Hip dysplasia, hindfoot pronation, scoliosis
8	M	1.4	nr	nr	TA type 2, RAA		Motor and language delay and mild axial hypotonia		Micrognathia, hypertelorism, long palpebral fissures, and low and depressed nasal bridge	

nr: not reported; VSD: ventricular septal defect; ASD: atrial septal defect; CoA: aortic coarctation; HLHS: hypoplastic left heart syndrome; BAV: bicuspid aortic valve; JIA: juvenile idiopathic arthritis; PFO: patent foramen ovale; TOF: tetralogy of Fallot; PDA: patent ductus arteriosus; TA: truncus arteriosus; RAA: right-sided aortic arch.

**Table 2:** Lymphocyte subsets of the patients.

	P1	P2	P3	P4	P5	P6	P7	P8
Age (years) *	21.5	5.8	13.6	22.8	14.8	7.7	16	1.6
Lymphocyte (×10^3^/µL)	1.27 *1.8 (0.9–4.5)*	0.92 *3.8 (2.3–6.1)*	1.72 *2.3 (1.3–3.2)*	1.45 *1.8 (0.9–4.5)*	1.20 *2.3 (1.3–3.2)*	1.44 *2.5 (1.7–3.4)*	1.1 *2.3 (1.3–3.2)*	2.89 *4.7 (3.9–6.1)*
T cells (×10^3^/µL)	0.89 *1.5 (0.78–3.0)*	0.46 *2.6 (1.6–3.7)*	0.94 *1.6 (0.95–2.3)*	0.91 *1.5 (0.78–3.0)*	0.77 *1.6 (0.95–2.3)*	0.78 *1.8 (1.2–2.6)*	0.69 *1.6 (0.95–2.3)*	1.27 *3.1 (2.5–4.9)*
Helper T cells (×10^3^/µL)	0.46 *1.0 (0.5–2.0)*	0.31 *1.4 (0.8–2.1)*	0.47 *0.9 (0.6–1.4)*	0.60 *1.0 (0.5–2.0)*	0.46 *0.9 (0.6–1.4)*	0.40 *1.0 (0.6–1.5)*	0.39 *0.9 (0.6–1.4)*	0.95 *1.8 (1.6–2.9)*
Cytotoxic T cells (×10^3^/µL)	0.36 *0.5 (0.2–1.2)*	0.14 *0.8 (0.4–1.1)*	0.41 *0.5 (0.3–0.7)*	0.17 *0.5 (0.2–1.2)*	0.21 *0.5 (0.3–0.7)*	0.22 *0.6 (0.3–0.9)*	0.28 *0.5 (0.3–0.7)*	0.17 *0.9 (0.6–1.4)*
B cells (×10^3^/µL)	0.18 *0.23 (0.06–0.8)*	0.27 *0.73 (0.4–1.2)*	0.37 *0.32 (0.2–0.7)*	0.21 *0.23 (0.06–0.8)*	0.20 *0.32 (0.2–0.7)*	0.21 *0.40 (0.3–0.6)*	0.94 *0.32 (0.2–0.7)*	1.03 *0.29 (0.19–0.7)*
NK cells (×10^3^/µL)	0.20 *0.34 (0.10–1.2)*	0.13 *0.29 (0.16–0.6)*	0.40 *0.23 (0.09–0.5)*	0.27 *0.34 (0.1–1.2)*	0.19 *0.23 (0.09–0.5)*	0.44 *0.26 (0.12–0.5)*	0.30 *0.23 (0.09–0.5)*	0.58 *0.29 (0.19–0.7)*

The absolute numbers of cell subsets are indicated for each patient (upper line). Lower lines indicate normal values for age (median (10–90th percentile)). * Age at immunological evaluation; NK: natural killer.

**Table 3:** Advanced phenotypes of the patients.

	P1	P2	P3	P4	P5	P6	P7	P8
Age (years) *	21.5	5.8	13.6	22.8	14.8	7.7	16	1.6
T cells (%) ^a^	69.1 *67 (50–91)*	50.2 *69 (60–77.6)*	54.6 *73 (62.6–80.4)*	62.5 *67 (50–91)*	64.3 *73 (62.6–80.4)*	54.0 *72 (63.2–77.8)*	63.0 *73 (62.6–80.4)*	44.0 *68 (60.7–75.8)*
Helper T cells (%) ^a^	36.5 *42 (28–64)*	33.2 *38 (31.1–47.4)*	27.2 *44 (32.6–51.5)*	41.7 *42 (28–64)*	38.4 *44 (32.6–51.5)*	27.5 *40 (31.7–47)*	28.1 *44(32.6–51.5)*	33.0 *41 (35–52)*
Cytotoxic T cells (%) ^a^	28.2 *22 (12–40)*	14.8 *21 (16–27)*	24.0 *23 (19–29)*	12.0 *22 (12–40)*	17.2 *23 (19–29)*	15.0 *24 (17.1–30)*	25.3 *23 (19–29)*	6.0 *19.3 (16.1–29.4)*
B cells (%) ^a^	13.8 *10 (4–28)*	29.0 *22 (13–29.2)*	21.5 *14 (12–21)*	14.3 *10 (4–28)*	16.3 *14 (12–21)*	14.5 *15.6 (12–34)*	8.5 *14 (12–21)*	35.5 *24 (14.3–28.2)*
NK cells (%) ^a^	15.5 *15 (4–24.6)*	14.0 *8 (4.7–16.2)*	23.5 *11.7 (4.3–16.2)*	18.3 *15 (4–24.6))*	15.5 *11.7 (4.3–16.2)*	30.7 *9.8 (5.4–18.6)*	27.6 *11.7 (4.3–16.2)*	20.0 *6.8 (4–13.8)*
Naïve helper T cells (%) ^b^	8.8 *46 (16–100)*	32.6 *70 (50–85)*	12.7 *51 (31–65)*	32.1 *46 (16–100)*	42.4 *51 (31–65)*	51.2 *58 (42–74)*	46.5 *51 (31–65)*	85.2 *79 (62–90)*
RTE (%) ^b^	2.7 *33 (7–100)*	13.0 *58 (41–81)*	2.9 *50 (31–81)*	20.6 *33 (7–100)*	27.0 *50 (31–81)*	26.6 *58 (41–81)*	41.0 *50 (31–81)*	39.9 *66 (40–100)*
CM helper T cells(%) ^b^	58.5 *42 (18–95)*	45.7 *18 (0.35–100)*	44.2 *32 (13–76)*	45.4 *42 (18–95)*	45.0 *32 (13–76)*	35.0 *18 (0.35–100)*	36.4 *32 (13–76)*	1.5 *10 (0.09–40)*
EM helper T cells (%) ^b^	32.5 *5 (1–23)*	20.0 *2 (0.27–18)*	39.0 *3 (0.49–25)*	22.0 *5 (1–23)*	12.2 *3 (0.49–25)*	12.7 *2 (0.27–18)*	16.8 *3 (0.49–25)*	1.4 *0.67 (0.024–4.7)*
TEMRA helper cells (%) ^b^	0.1 *0.35 (0.008–6.8)*	1.6 *0.1 (0.003–1.8)*	4.1 *0.17 (0.004–5.8)*	0.6 *0.35 (0.008–6.8)*	0.3 *0.17 (0.004–5.8)*	1.1 *0.1 (0.003–1.8)*	0.3 *0.17 (0.004–5.8)*	11.9 *0.1 (0.0–4.1)*
Naïve cytotoxic T cells (%) ^c^	10.5 *29 (6–100)*	53.9 *64 (42–81)*	9.0 *56 (42–73)*	17.8 *29 (6–100)*	78.8 *56 (42–73)*	30.6 *58 (39–73)*	75.2 *56 (42–73)*	73.1 *71(46–85)*
CM cytotoxic T cells (%) ^c^	35.2 *5 (1–20)*	4.0 *3 (1–6)*	10.4 *3 (0.4–18)*	17.7 *5 (1–20)*	10.3 *3 (0.4–18)*	10.7 *3 (1–6)*	24.8 *3 (0.4–18)*	0.5 *3 (1–8)*
EM cytotoxic T cells (%) ^c^	47.4 *36 (14–98)*	6.1 *24 (5–100)*	33.2 *22 (4–100)*	13.3 *36 (14–98)*	3.3 *22 (4–100)*	35.3 *24 (5–100)*	0.1 *22 (4–100)*	2.1 *15 (2–100)*
TEMRA cytotoxic T cells (%) ^c^	6.8 *19 (7–53)*	22.8 *25 (15–41)*	47.3 *24 (9–65)*	17.8 *19 (7–53)*	7.6 *24 (9–65)*	23.3 *25 (15–41)*	0.10 *24 (9–65)*	24.3 *24 (8–71)*
Treg (%) ^b^	10 *8 (4–17)*	15.8 *8 (4–14)*	4.4 *9 (4–20)*	8.6 *8 (4–17)*	9.8 *9 (4–20)*	3.4 *8 (4–14)*	13.2 *9 (4–20)*	6.2 *9 (6–13)*
Follicular T helper cells (%) ^d^	27.4 *17 (5–56)*	36.5 *24 (7–85)*	22.7 *18 (7–47)*	25.9 *17 (5–56)*	26.2 *18 (7–47)*	44.8 *24 (7–85)*	28.6 *18 (7–47)*	27.1 *20 (8–51)*
Naïve B cells (%) ^e^	53.8 *63 (33–100)*	82.0 *76 (62–94)*	91.4 *74 (49–100)*	78.1 *63 (33–100)*	89.1 *74 (49–100)*	61.9 *76 (62–94)*	47.2 *74 (49–100)*	97.4 *88 (78–99)*
Switched memory B cells (%) ^e^	14.6 *12 (3–46)*	4.0 *7 (3–18)*	2.0 *8 (1–43)*	7.2 *12 (3–46)*	2.5 *8 (1–43)*	24.0 *7 (3–18)*	30.4 *8 (1–43)*	1.04 *3 (0.3–20)*

The frequency of cell subsets is indicated for each patient (upper line). Lower lines indicate normal values for age (median (10–90th percentile)). * Age at immunological evaluation. ^a^ % of total peripheral lymphocyte population; ^b^ % of helper T lymphocyte population; ^c^ % of cytotoxic T lymphocyte population; ^d^ % of CD4^+^CD45RO^+^ T lymphocytes; ^e^ % of B lymphocyte population; NK: natural killer; TEMRA: effector memory T cells re-expressing CD45RA; CM: central memory; EM effector memory; RTE: recent thymic emigrants; Treg: regulatory T cells.

**Table 4:** Absolute and relative numbers of the dendritic cells in the cohort.

	P1	P2	P3	P4	P5	P6	P7	P8
Age (years) *	21.5	5.8	13.6	22.8	14.8	7.7	16	1.6
DCtot/µL	31.71 *24 (10.7–35.6)*	10.69 *41.4 (28.6–69.5)*	26.41 *36 (27.1–43.7)*	18.15 *24 (10.7–35.6)*	20.74 *36 (27.1–43.7)*	32.61 *41.4 (28.6–69.5)*	14.3 *36 (27.1–43.7)*	59.07 *53.6 (44.7–58.9)*
DCtot (%) ^f^	0.48 *0.43 (0.22–0.69)*	0.25 *0.56 (0.39–0.68)*	0.56 *0.61 (0.5–0.72)*	0.44 *0.43 (0.22–0.69)*	0.46 *0.61 (0.5–0.72)*	0.72 *0.56 (0.39–0.68)*	0.23 *0.61 (0.5–0.72)*	1.12 *0.63 (0.48–0.89)*
mDC/µL	18.29 *14.7 (7.6–21.1)*	7.33 *25.5 (12.4–48.0)*	17.98 *23.5 (18.4–30.9)*	11.37 *14.7 (7.6–21.1)*	11.36 *23.5 (18.4–30.9)*	14.6 *25.5 (12.4–48.0)*	5.69 *23.5 (18.4–30.9)*	27.62 *32.8 (26.9–39.5)*
mDC (%) ^f^	0.28 *0.26 (0.1–0.4)*	0.17 *0.34 (0.2–0.5)*	0.38 *0.40 (0.3–0.5)*	0.28 *0.26 (0.1–0.4)*	0.25 *0.40 (0.3–0.5)*	0.32 *0.34 (0.2–0.5)*	0.09 *0.40 (0.3–0.5)*	*0.52* *0.38 (0.3–0.5)*
pDC/µL	13.42 *9.4 (3.2–17.0)*	3.36 *15.9 (8.6–23.6)*	8.43 *12.5 (5.4–18.8)*	6.78 *9.4 (3.25–17.0)*	9.38 *12.5 (5.4–18.8)*	18.01 *15.9 (8.6–23.6)*	8.61 *12.5 (5.4–18.8)*	31.45 *20.8 (12.6–30.8)*
pDC (%) ^f^	0.20 *0.17 (0.07–0.3)*	0.08 *0.22 (0.1–0.4)*	0.18 *0.21 (0.1–0.3)*	0.16 *0.17 (0.07–0.3)*	0.21 *0.21 (0.1–0.3)*	0.4 *0.22 (0.1–0.4)*	0.14 *0.21 (0.1–0.3)*	0.6 *0.25 (0.1–0.4)*

The frequency and absolute numbers of cell subsets are indicated for each patient (upper line). Lower lines indicate normal values for age (mean (10–90th percentile)). * Age at immunological evaluation; ^f^ % of WBC; WBC: white blood cells; DC: dendritic cells; mDC: myeloid dendritic cells; pDC: plasmacytoid dendritic cells.

**Table 5:** Immunoglobulins and their subclasses in the cohort.

Immunoglobulins	P1	P2	P3	P4	P5	P6	P7	P8
Age *	21.5	5.8	13.6	22.8	14.8	7.7	16	1.6
IgG (mg/dL)	1440 *(1116 ± 208)*	979 *(1007 ± 236)*	841 *(1116 ± 208)*	557 *(1116 ± 208)*	697 *(1116 ± 208)*	1000 *(1040 ± 223)*	962 *(1116 ± 208)*	361 *(655 ± 176)*
IgM (mg/dL)	256 *(92 ± 34)*	57 *(87 ± 27)*	96 *(92 ± 34)*	304 *(92 ± 34)*	91 *(92 ± 34)*	49 *(90 ± 27)*	127 *(92 ± 34)*	21 *(67 ± 29)*
IgA (mg/dL)	138 *(189 ± 67)*	84 *(123 ± 41)*	113 *(189 ± 67)*	59 *(189 ± 67)*	48 *(189 ± 67)*	163 *(136 ± 48)*	155 *(189 ± 67)*	35 *(42 ± 23)*
IgG1 (mg/dL)	844 *(490–1140)*	664 *(370–1000)*	NA	523 *(490–1140)*	535 *(490–1140)*	730 *(370–1000)*	588 *(370–1280)*	310 *(200–770)*
IgG2 (mg/dL)	511 *(150–640)*	181 *(72–340)*	NA	196 *(150–640)*	152 *(150–640)*	213 *(72–340)*	266 *(106–610)*	83 *(34–230)*
IgG3 (mg/dL)	64 *(20–110)*	77 *(13–133)*	NA	23 *(20–110)*	30 *(20–110)*	59 *(13–133)*	57 *(18–263)*	13 *(15–97)*
IgG4(mg/dL)	93 *(8–140)*	2 *(0.01–158)*	NA	0.0 *(8–140)*	9 *(8–140)*	24 *(0.01–158)*	25 *(4–230)*	2 *(0.01–43)*

The frequency of immunoglobulins and IgG subclasses is indicated for each patient (upper line). Lower lines indicate normal values for age expressed as mean ± SD or 10–90th percentile; NA, not available. * Age at immunological evaluation.

**Table 6:** Genetic abnormalities on array-CGH of the cohort.

Subjects	Position (GRCh37/hg19)	Extent (kb)	NCBI RefSeq Genes (UCSC)	Inheritance
P1	2q24.1 (156,761,199_157,075,778)x3	314	LINC01876	Maternal
P2	arr(X,Y)x1,(1-22)x2 *			
P3	arr(X,Y)x1,(1-22)x2 *			
P4	20p11.22 (21,419,411_21,784,484)x3	365	**PAX1**, NKX2-2, LINC01727,LINC01726	Maternal
P5	20p11.22 (21,419,411_21,784,484)x3	365	**PAX1**, NKX2-2, LINC01727,LINC01726	Maternal
P6	11p15.5 (723,382_917,649)x3	194	**EPS8L2**, **TALD01**, GATD1, LOC171391, CEND1, **SLC25A22**, PIDD1, RPLP2, SNORA52, **PNPLA2**, CRACR2B, **CD151**, POLR2L, TSPAN4, CHID1	NA
	Xp22.33 or Yp11.32 (61,091_658,258 or 11,091_608,258)x2	597	PLCXD1, GTPBP6, LINC00685, PPP2R3B, **SHOX**	
P7	17q21.31 (43,717,703_44,210,822)x1	493	LINC02210, LINC02210-CRHR1, CRHR1, MAPT-AS1, SPPL2C, **MAPT**, MAPT-TT1, STH, **KANSL1**	NA
P8	17p13.2(5882589_6140992)x1	258	WSCD1	Maternal
	Xq24(118647205_118715504)x0	68	**CXorf56, UBE2A**	

OMIM genes are in bold; NA: not available; * negative array-CGH.

## Data Availability

The data presented in this study are available on reasonable request from the corresponding author.

## Supplementary Materials

The following supporting information can be downloaded at https://www.mdpi.com/article/10.3390/jcm11072025/s1, Table S1: Gene content in the CNVs; Figure S1: Auxological findings of height expressed in standard deviations.
