# Supplementary material for: Clinical, Immunological, and Genetic Findings in a Cohort of Patients with the DiGeorge Phenotype without 22q11.2 Deletion
Source: J Clin Med. 2022 Apr 5;11(7):2025. doi: 10.3390/jcm11072025 (PMC8999496; doi:10.3390/jcm11072025)
Supplement: Supplementary file 1 [file jcm-11-02025-s001.zip › jcm-1635510-supplementary.pdf]

Table S1: Gene content in the CNVs

**2q24.1(156,761,199-157,075,778)x3**			
**NCBI**	**Description**	**OMIM**	**pLI**
LINC01876	long intergenic non-protein coding RNA 1876		/
**20p11.22(21,419,411-21,784,484)x3**			
**NCBI**	**Description**	**OMIM**	**pLI**
NKX2-2	NK2 homeobox 2		0.41
LINC01727	long intergenic non-protein coding RNA 1727		/
LINC01726	long intergenic non-protein coding RNA 1726		/
**PAX1** (*167411)	Paired box 1	(#615560) Otofaciocervical syndrome 2 (AR)	0.70
**11p15.5(723,382-917,649)x3**			
**NCBI**	**Description**	**OMIM**	**pLI**
**EPS8L2** (*614988)	EPS8 like 2	(#617637) Deafness autosomal recessive 106 (AR)	0.00
**TALD01** (*602063)	Transaldolase 1	(#606003) Transaldolase deficiency (AR)	0.00
GATD1 (NCBI)	Glutamine amidotransferase like class 1 domain containing 1		0.46
LOC171391	uncharacterized LOC171391		/
CEND1	Cell cycle exit and neuronal differentiation 1		0.60
**SLC25A22** (*609302)	Solute carrier family 25 (mitochondrial carrier, glutamate), member 22	(#609304) Developmental and epileptic encephalopathy 3 (AR)	0.01
PIDD1	p53-induced death domain protein 1		0.00
RPLP2	Ribosomal protein lateral stalk subunit P2		0.67
SNORA52	small nucleolar RNA, H/ACA box 52		/
**PNPLA2** (*609059)	Patatin-like phospholipase domain-containing protein 2	(#610717) Neutral lipid storage disease with myopathy (AR)	0.00
CRACR2B	Calcium release activated channel regulator 2B		0.00
**CD151** (*602243)	CD151 molecule (Raph blood group)	(#609057) Epidermolysis bullosa simplex 7, with nephropathy and deafness<br>(#179620) [Blood group, Raph]	0.00
POLR2L	RNA polymerase II, I and III subunit L		0.00

TSPAN4	Tetraspanin 4		0.00
CHID1	Chitinase domain containing 1		0.00
**Xp22.33 or Yp11.32 (61,091-658,258 or 11,091-608,258)x2**			
**NCBI**	**Description**	**OMIM**	**pLI**
PLCXD1	Phosphatidylinositol specific phospholipase C X domain containing 1		0.00
GTPBP6	GTP binding protein 6 (putative)		0.00
LINC00685	long intergenic non-protein coding RNA 685		/
PPP2R3B	Protein phosphatase 2 regulatory subunit B"beta		0.00
**SHOX** (*312865/*400020)	Short stature homeobox	(#249700) Langer mesomelic dysplasia (PR)	0.67
		(#127300) Leri-Weill dyschondrosteosis (PD)	
		(#300582) Short stature, idiopathic familial	
**17q21.31(43,717,703-44,210,822)x1 / syndrome Koolen de Vries 17q21.31(43,705,166 - 44,294,406)x1 (Decipher)**			
**NCBI**	**Description**	**OMIM**	**pLI**
LINC02210			/
LINC02210-CRHR1			/
CRHR1			0.00
MAPT-AS1			/
SPPL2C			0.00
**MAPT** (*157140)	Microtubule associated protein tau	(#600274) Dementia, frontotemporal, with or without parkinsonism (AD)	0.00
		(#172700) Pick disease (AD)	
		(#601104) Supranuclear palsy, progressive (AD)	
		(#260540) Supranuclear palsy, progressive atypical (AR)	
		(#168600) {Parkinson disease, susceptibility to} (AD, multifactorial)	
MAPT-TT1	MAPT intronic transcript 1		/
STH	Saitohin		/
**KANSL1** (*612452)	KAT8 regulatory NSL complex subunit 1	(#610443) Koolen-De Vries syndrome (AD)	1.00

**17p13.2(5882589_6140992)x1**			
**NCBI**	**Description**	**OMIM**	**pLI**
WSCD1	WSC domain containing 1		0.00
**Xq24(118647205_118715504)x0**			
**NCBI**	**Description**	**OMIM**	**pLI**
**CXorf56** (*301012)	STEEP1 - STING1 ER exit protein 1	(#301013) Intellectual developmental disorder, X-linked 107 (X-linked)	0.91
**UBE2A** (*312180)	UBE2A - Ubiquitin-conjugating enzyme E2A	(#300860) Intellectual developmental disorder, X-linked syndromic, Nascimento type (X-linked recessive)	0.81

AR: Autosomal recessive; PR: Pseudoautosomal recessive; PD: Pseudoautosomal dominant; AD: Autosomal dominant; pLI: probability of loss of function intolerance; green: non coding protein genes.

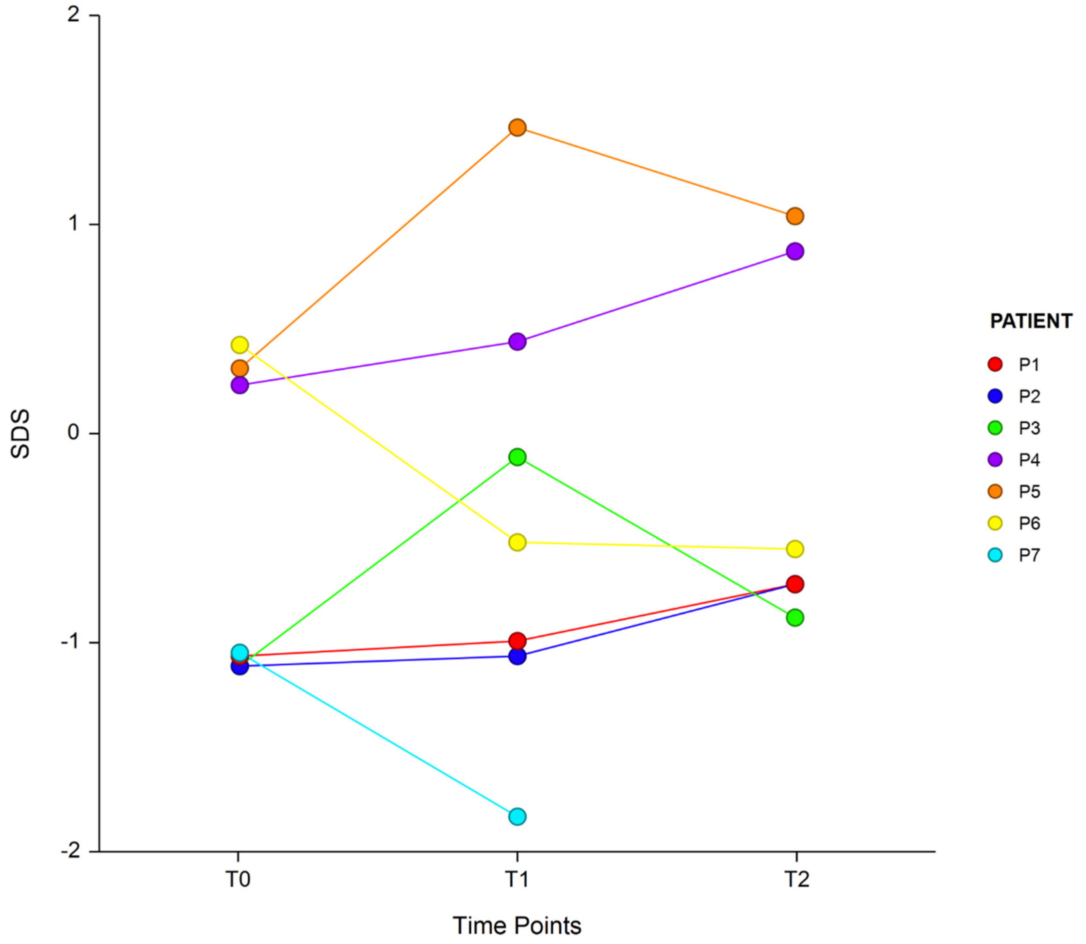

Time point	Height (SDS) Median	IQR
T0	-1.05	1.42
T1	-0.52	1.50
T2	-0.64	1.67

Figure S1: Auxological findings of height expressed in standard deviations.
